# Prevalence of *Plasmodium falciparum* lacking histidine-rich proteins 2 and 3: a systematic review

**DOI:** 10.2471/BLT.20.250621

**Published:** 2020-06-19

**Authors:** Rebecca Thomson, Jonathan B Parr, Qin Cheng, Stella Chenet, Mark Perkins, Jane Cunningham

**Affiliations:** a London, England.; b Division of Infectious Diseases, University of North Carolina, Chapel Hill, United States of America.; c Australian Defence Force Malaria and Infectious Disease Institute, Queensland, Australia.; d Instituto de Enfermedades Tropicales, Universidad Nacional Toribio Rodríguez de Mendoza de Amazonas, Chachapoyas, Peru.; e Department of Emergency Preparedness, World Health Organization, Geneva, Switzerland.; f Global Malaria Programme, World Health Organization, avenue Appia 20, 1211 Geneva 27, Switzerland.

## Abstract

**Objective:**

To calculate prevalence estimates and evaluate the quality of studies reporting *Plasmodium falciparum* lacking histidine-rich proteins 2 and 3, to inform an international response plan.

**Methods:**

We searched five online databases, without language restriction, for articles reporting original data on *Plasmodium falciparum*-infected patients with deletions of the *pfhrp2* and/or *pfhrp3* genes (*pfhrp2/3*). We calculated prevalence estimates of *pfhrp2/3* deletions and mapped the data by country. The denominator was all *P. falciparum*-positive samples testing positive by microscopy and confirmed positive by species-specific polymerase chain reaction testing (PCR). If microscopy was not performed, we used the number of samples based on a different diagnostic method or PCR alone. We scored studies for risk of bias and the quality of laboratory methods using a standardized scoring system.

**Findings:**

A total of 38 articles reporting 55 studies from 32 countries and one territory worldwide were included in the review. We found considerable heterogeneity in the populations studied, methods used and estimated prevalence of *P. falciparum* parasites with *pfhrp2/3* deletions. The derived prevalence of *pfhrp2* deletions ranged from 0% to 100%, including focal areas in South America and Africa. Only three studies (5%) fulfilled all seven criteria for study quality.

**Conclusion:**

The lack of representative surveys or consistency in study design impairs evaluations of the risk of false-negative results in malaria diagnosis due to *pfhrp2/3* deletions. Accurate mapping and strengthened monitoring of the prevalence of *pfhrp2/3* deletions is needed, along with harmonized methods that facilitate comparisons across studies.

## Résumé

**Objectif:**

Estimer la prévalence et évaluer la qualité des études consacrées à *Plasmodium falciparum* sans protéines 2 et 3 riches en histidine afin d'établir un plan d'intervention international.

**Méthodes:**

Nous avons parcouru cinq bases de données en ligne sans restriction de langue pour trouver des articles contenant des informations d'origine relatives à des patients atteints de *Plasmodium falciparum* dépourvu des gènes *pfhrp2* et/ou *pfhrp3* (*pfhrp2/3*). Nous avons calculé la prévalence des délétions des gènes *pfhrp2/3* et cartographié les données par pays. Le dénominateur était représenté par les échantillons positifs à *P. falciparum*, testés positifs au microscope et confirmés par un test de réaction en chaîne par polymérase (PCR) propre à l'espèce. Si aucun examen n'avait été effectué au microscope, nous avons utilisé le nombre d'échantillons recourant à une méthode de diagnostic différente, ou uniquement à la PCR. Nous avons noté les études selon le risque de biais et la qualité des techniques d'analyse en laboratoire, à l'aide d'un système de notation standardisé.

**Résultats:**

Au total, 38 articles mentionnant 55 études réalisées dans 32 pays et un territoire dans le monde ont été pris en compte dans cette revue. Nous avons observé une grande hétérogénéité dans les populations étudiées, les méthodes employées et la prévalence estimée des parasites *P. falciparum* assortis d'une délétion des gènes *pfhrp2/3*. La prévalence dérivée des délétions de *pfhrp2* est comprise entre 0% et 100%, avec des zones de convergence en Amérique du Sud et en Afrique. Seules trois études (5%) remplissaient l'ensemble des sept critères de qualité.

**Conclusion:**

L'absence d'enquêtes représentatives ou d'uniformité dans la conception des études empêche d’évaluer correctement le risque de faux négatifs dans le diagnostic de la malaria en raison des délétions de *pfhrp2/3*. Une cartographie détaillée ainsi qu'une surveillance renforcée de la prévalence des délétions de *pfhrp2/3* est nécessaire, tout comme une harmonisation des méthodes afin de faciliter la comparaison entre les différentes études.

## Resumen

**Objetivo:**

Calcular las estimaciones de la prevalencia y evaluar la calidad de los estudios que informan de la existencia del *Plasmodium falciparum* que carece de las proteínas 2 y 3 ricas en histidina, para elaborar un plan de respuesta internacional.

**Métodos:**

Se revisaron cinco bases de datos en línea, sin restricción de idioma, para encontrar artículos que informaran sobre los datos originales de los pacientes infectados con *Plasmodium falciparum* con deleciones de los genes *pfhrp2* y/o *pfhrp3* (*pfhrp2/3*). Se calcularon las estimaciones de prevalencia de las deleciones de *pfhrp2/3* y se clasificaron los datos por país. El denominador eran todas las muestras positivas por *P. falciparum* que daban positivo en las pruebas de microscopía y confirmadas como positivas en las pruebas de reacción en cadena de la polimerasa (PCR, por sus siglas en inglés) específicas de la especie. Si no se realizaba la microscopía, se empleaba el número de muestras en base a un método de diagnóstico diferente o a la PCR únicamente. Los estudios se calificaron en función del riesgo de sesgo y de la calidad de los métodos de laboratorio por medio de un sistema de puntuación estandarizado.

**Resultados:**

El análisis incluyó un total de 38 artículos en los que se informaba de 55 estudios de 32 países y un territorio a nivel mundial. Se observó una heterogeneidad considerable en las poblaciones estudiadas, los métodos aplicados y la prevalencia estimada de los parásitos *P. falciparum* con deleciones de los genes *pfhrp2/3*. La prevalencia que se estimó de las deleciones del gen *pfhrp2* osciló entre el 0 % y el 100 %, incluidas las áreas focales de América del Sur y África. Tan solo tres estudios (5 %) cumplieron los siete criterios de calidad del estudio.

**Conclusión:**

La falta de encuestas fiables o de consistencia en el diseño de los estudios dificulta las evaluaciones del riesgo de resultados falsos negativos en el diagnóstico de la malaria debido a las deleciones de los genes *pfhrp2/3*. Se necesita un mapeo preciso y un seguimiento reforzado de la prevalencia de las deleciones de los genes *pfhrp2/3*, junto con métodos estandarizados que faciliten las comparaciones entre los estudios.

## ملخص

الغرض حساب تقديرات الانتشار، وتقييم جودة الدراسات التي تشير إلى المتصورة المنجلية (الملاريا الخبيثة *) * التي تفتقر إلى البروتينات الغنية بالهيستدين 2 و3، وذلك لوضع خطة الاستجابة الدولية.

الطريقة قمنا بالبحث في خمس قواعد للبيانات على الإنترنت، دون التقيد بلغة، عن المقالات التي تحتوي على بيانات أصلية عن المرضى المصابين بالمتصورة المنجلية، مع حذف جينات pfhrp2 و/أو pfhrp3 (pfhrp2/3). قمنا بحساب تقديرات الانتشار لحالات حذف pfhrp2/3، وقمنا بتصنيف البيانات حسب البلد. كان القاسم المشترك هو جميع العينات الإيجابية للمتصورة المنجلية، التي كانت نتيجتها إيجابية بالفحص المجهري، وتأكد أنها إيجابية بواسطة اختبار تفاعل سلسلة بوليميريز النوعي (PCR). إذا لم يتم إجراء الفحص المجهري، فإننا استخدمنا عدد العينات بناءً على طريقة تشخيص مختلفة أو اختبار PCR بمفرده. قمنا بتقييم دراسات لمخاطر التحيز، وجودة طرق المختبر باستخدام نظام تقييم قياسي.

النتائج تضمنت المراجعة إجمالي 38 مقالاً عن 55 دراسة من 32 دولة، ومنطقة واحدة حول العالم. اكتشفنا وجود عدم تجانس ملموس في السكان الذين خضعوا للدراسة، والطرق المستخدمة، والانتشار التقديري لطفيليات المتصورة المنجلية مع حالات حذف pfhrp2/3. تراوح الانتشار المترتب لحالات حذف pfhrp2 من 0% إلى 100%، بما في ذلك المناطق البؤرية في أمريكا الجنوبية وأفريقيا. ثلاث دراسات فقط (5%) استوفت كل المعايير السبعة لجودة الدراسة.

الاستنتاج أدى النقص في الاستقصاءات التمثيلية، أو الاتساق في تصميم الدراسة، إلى إضعاف تقييمات مخاطر النتائج السلبية الكاذبة في تشخيص الملاريا بسبب حالات حذف pfhrp2/3. هناك حاجة للتصنيف الدقيق والمراقبة المدعمة لانتشار حالات حذف pfhrp2/3، إلى جانب الأساليب المتناغمة التي تسهل المقارنات بين الدراسات. ****

## 摘要

**目的:**

旨在计算感染率估值并评估报告缺失富组氨酸蛋白 2 和 3 的*恶性疟原虫*的研究质量，以制定一项国际性应对计划。

**方法:**

我们在没有语言限制的情况下搜索了五个在线数据库，以获取报告缺失 *pfhrp2* 和/或 *pfhrp3* 基因 (*pfhrp2/3*) 的*恶性疟原虫*感染患者原始数据的文章。我们计算了缺失 *pfhrp2/3* 的感染率估值并按国家映射数据。分母是所有经显微镜检测均为阳性并通过种特异性聚合酶链反应试验 (PCR) 确认为阳性的*恶性疟原虫*阳性样本。如果进行显微镜检测，我们采用基于不同诊断方法或单独 PCR 的样本数量。我们使用标准化评分系统对偏倚风险以及实验室方法的质量研究进行了评分。

**结果:**

评审中共纳入 38 篇文章，这些文章报告了全球 32 个国家和 1 个地区中的 55 项研究。在研究的人群、使用的方法以及估计的缺失 *pfhrp2/3 *的*恶性疟原虫*感染率中，我们发现了相当大的异质性。推导的 *pfhrp2* 缺失感染率介于 0％ 至 100％ 之间，包括南美洲和非洲的重点地区。就研究质量而言，只有三项研究 (5%) 符合所有七个标准。

**结论:**

缺乏代表性调查或研究设计缺乏一致性妨碍了由于 *pfhrp2/3* 缺失导致疟疾诊断中假阴性结果风险的评估。需要准确映射和加强对 *pfhrp2/3* 缺失感染率的监测，以及促进各项研究比较的统一方法。

## Резюме

**Цель:**

Вычислить распространенность и оценить качество исследований, посвященных распространенности *Plasmodium falciparum* с отсутствием богатых гистидином белков 2 и 3, с целью получения информации для разработки международного плана реагирования.

**Методы:**

Авторы выполнили поиск статей, содержащих исходные данные по пациентам, зараженным *Plasmodium falciparum* с делецией генов *pfhrp2* и/или *pfhrp3* (*pfhrp2/3*), в пяти базах данных в Интернете на разных языках. Авторы выполнили оценку распространенности делеций *pfhrp2/3* и составили карту данных для разных стран. В качестве знаменателя использовалось общее количество проб на *P. falciparum* с положительным результатом, полученным при микроскопическом исследовании и подтвержденным в ходе видоспецифичного тестирования методом полимеразной цепной реакции (ПЦР). Если микроскопическое исследование не проводилось, авторы использовали данные о количестве проб, полученные на основании другого диагностического метода или только на основании ПЦР. Исследования оценивались по уровню риска необъективности и качеству лабораторных методов с применением стандартной системы оценок.

**Результаты:**

В общей сложности в обзор были включены 38 статей, содержащих сведения о 55 исследованиях, проведенных в 32 странах мира и на одной территории. Авторы обнаружили значительную неоднородность в исследованных популяциях, использованных методах и оценке распространенности паразитов *P. falciparum* с делециями *pfhrp2/3*. Производное значение распространенности делеций *pfhrp2* находилось в диапазоне от 0 до 100%, включая очаговые области в Южной Америке и Африке. Только три исследования (5%) соответствовали всем семи критериям качества для исследований.

**Вывод:**

Отсутствие репрезентативных исследований или недостаточное единообразие планов исследований отрицательно влияют на оценку риска ложноотрицательных результатов при диагностировании малярии по причине делеций *pfhrp2/3*. Необходимы точное отображение данных и усиленный мониторинг распространенности делеций *pfhrp2/3*, а также разработка гармонизированных методов, которые упростят сравнение разных исследований между собой.

## Introduction

Despite improvements in malaria control over the past decade, malaria caused an estimated 405 000 deaths worldwide in 2018.^1^ In 2010, World Health Organization (WHO) treatment guidelines established that all cases of suspected malaria should be confirmed by microscopy or an antigen-detecting rapid diagnostic test before treatment.^2^ Malaria rapid diagnostic tests contain one or a combination of antibodies that recognize specific plasmodial antigens. These antigens include histidine-rich protein 2 (HRP2) which is specific to *P. falciparum*, and genus- and species-specific lactate dehydrogenase or aldolase, which are produced by all four major human-infecting *Plasmodium* species.^3^ The number of rapid diagnostic tests procured has increased significantly, from 10 million in 2002 to 412 million in 2018.^1^ The great majority of these tests detect an HRP2 target, alone or with another antigen, with 15 of 16 (94%) WHO-prequalified malaria tests targeting HRP2 for *P. falciparum* detection.^4^

Rapid diagnostic tests targeting HRP2 came to dominate the market because they are generally more sensitive than other assays and tend to be more heat stable.^5^^,^^6^ The presence of repetitive epitopes in HRP2 provides numerous antibody binding sites and enables the detection of low levels of protein. The monoclonal antibodies used in HRP2-detecting tests often cross-react with HRP3, encoded by the *pfhrp3* gene,^7^^,^^8^ particularly at parasite counts above 1000 per µL of blood.^9^ HRP3 is a structural homologue of HRP2 that shares similar amino-acid repeats.^8^^,^^10^

Deletions in the *pfhrp2* and/or *pfhrp3* (*pfhrp2/3*) genes as a cause of false-negative rapid diagnostic tests was first recognized in 2010 in the Peruvian Amazon basin.^11^ Molecular testing by polymerase chain reaction (PCR) confirmed *P. falciparum* infection, but also that *pfhrp2* and *pfhrp3* genes were deleted in 41% (61 samples) and 70% (103 samples) of these 148 samples, respectively.^11^ Additional analyses have confirmed a significant increase in the frequency of samples showing *pfhrp2/3* deletions in the same area.^12^^,^^13^ More recently, malaria parasites with *pfhrp2/3* gene deletions have been documented in other parts of the world including East,^9^^,^^14^ Central,^15^ West^16^ and Southern Africa,^17^ Asia^18^ and the Middle East.^19^ Most concerning was a study in Eritrea that reported samples from 62% (31/50) of microscopy-confirmed *P. falciparum* patients testing negative for *pfhrp2*.^20^ Collectively, these reports suggest a global threat to the continued use of HRP2-based rapid diagnostic tests.

In 2014, recommendations on investigating and accurate reporting of *pfhrp2/3* gene deletions were published.^21^ Additional criteria have been proposed in more recent studies, including parasite quantification by microscopy or quantitative PCR to rule out false-negative *pfhrp2* detection in samples below the limit of detection of the *pfhrp2* assay,^9^ and analysis of *pfhrp3*.^22^ However, we have found no assessments of the uptake of these recommendations.

There are increasing numbers of reports documenting the threat of mutant parasite genes for malaria case management. However, due to different study designs and laboratory methods it is difficult to compare findings across studies and accurately understand this threat. We aimed to compile all published studies on the prevalence of *pfhrp2/3* gene deletions and assess the quality of methods and reporting. We used our findings to paint a global picture of the current status of *pfhrp2/3* deletions to guide decisions on the locations and methods of future surveys.

## Methods

### Search strategy and data extraction

We carried out a systematic review according to the Preferred Reporting Items for Systematic Reviews and Meta-analyses statement.^23^ We made a search of the online databases of PubMed®, Scopus, LILACS (Literatura Latino-Americana e do Caribe em Ciências da Saúde), WHO Global Index Medicus and the Web of Science for articles published in any language between 1 January 2010 and 20 August 2019. We used the search terms “[histidine* OR hrp* OR pfhrp*] AND [deletion* OR variation OR diversity OR lack] AND [malaria OR falciparum]” to identify articles reporting molecular analysis of *P. falciparum* parasite samples for *pfhrp2/3* deletions. Additional articles were identified through manual searches. Further information about the search criteria are provided in Table 1. Two investigators screened the titles and abstracts of all eligible articles and extracted the following information from the full text: country, study sites, study design, year(s) of data collection, patient symptom status, age range, number of *P. falciparum*-positive patients, type of blood sample, which samples underwent molecular analysis, number of samples with *pfhrp2*/*3* deletions, laboratory methods (seven items; Box 1) and analysis of flanking genes. Discrepancies in the data were double-checked.

**Table 1:** Inclusion and exclusion criteria for selection of studies in the systematic review of *Plasmodium falciparum pfhrp2/3* gene deletions

Characteristic	Included	Excluded
Study population	All ages and populations	None
Study outcome	Percentage of samples testing negative for *pfhrp2* gene, with or without analysis of *pfhrp3* gene	Studies which analysed variation in *pfhrp2* genetic sequence only
Method of confirmation of *pfhrp2* and/or *pfhrp3* gene deletion	Molecular analysis of *pfhrp2/3* gene deletions	Suspected deletions based on rapid diagnostic testing, microscopy or serological testing only
Study design	All, including case studies, cross-sectional or convenience studies	None
Type of paper	Published articles of an original study	Review articles, doctoral theses, abstracts with no corresponding published article
Patient status	Symptomatic suspected malaria patients or asymptomatic people	None
Area of data collection	All countries and regions	None
Date of study publication	1 January 2010 to 20 August 2019	Prior to 1 January 2010

Box 1 **Assessment of study quality in the systematic review of *Plasmodium falciparum pfhrp2/3* gene deletions**

We assessed a total of seven criteria for quality of laboratory methods, five based on recommendations from previous reasearch^21^ and two additional criteria.^9^^,^^22^ The number and percentage of studies complying with each quality criterion were as follows (*n* = 55 studies):

- Quality-assured microscopy: 45 studies (82%).
- *Plasmodium falciparum* species confirmation by PCR test: 55 studies (100%).
- Detection of two other single-copy genes: 21 studies (38%).
- HRP2 detection by serological analysis or using a second brand of WHO-prequalified HRP2-detecting rapid diagnostic test: 13 studies (24%).
- Detection of *pfhrp3* gene by PCR test: 46 studies (84%).
- Use of WHO-prequalified rapid diagnostic test: 27 studies (49%).
- Parasite density quantification^:^ 36 studies (66%).

We awarded one point per criterion satisfied and assigned a total quality score for each study (from 1 to 7), as follows:

Score 1: 0 studies (0%); Score 2: 6 studies (11%); Score 3: 5 studies (9%); Score 4: 16 studies (30%); Score 5: 15 studies (27%); Score 6: 10 studies (18%); Score 7: 3 studies (6%).

HRP2: histidine-rich protein 2; PCR: polymerase chain reaction; WHO: World Health Organization.

### Prevalence estimates

To maximize consistency in calculating prevalence across studies, we used the total number of *P. falciparum* samples testing positive by microscopy and confirmed *P. falciparum*-positive by species-specific PCR as the denominator. We did this regardless of whether all or only a subset of patient samples were tested for *pfhrp2/3* deletion by molecular analysis or whether it was the denominator reported in the original publication. If microscopy was not performed, we used the number of samples based on a different diagnostic method or PCR alone. We did not make prevalence estimates from case reports. All prevalence estimates in this review were therefore derived using a standardized denominator and not necessarily the same prevalence as reported in the original article.

Where researchers collected samples from multiple countries, or used different sampling methods or time frames, we separated the results by country or sample collection group to present prevalence data as separate studies. We presented compiled results for studies which collected samples at one point in time from multiple sites with the same sampling design. When we combined data from different studies by country, we weighted the percentage of samples with *pfhrp2* gene deletions to account for differing sample sizes. We used the middle year of the data collection period for studies spanning multiple years.

### Assessment of study quality and bias

We assigned a total quality score from 1 to 7 to each study, based on fulfilment of seven criteria for quality of laboratory methods (Box 1).

We assessed study bias as a score from 1 (lowest bias) to 4 (highest bias). The values show the potential bias of the derived prevalence estimate from the true prevalence in the population, depending on the sample population (symptomatic, asymptomatic, mixed or unrepresentative) and samples tested for *pfhpr2/3* genes (all, discordant only or another subset). Studies which analysed all samples have a lower bias score than those which only analysed discordant or a subset of samples, while studies which included both symptomatic and asymptomatic samples have a lower study bias than those which only analysed samples from symptomatic people or an unrepresentative sample. 

## Results

**Fig. 1:**
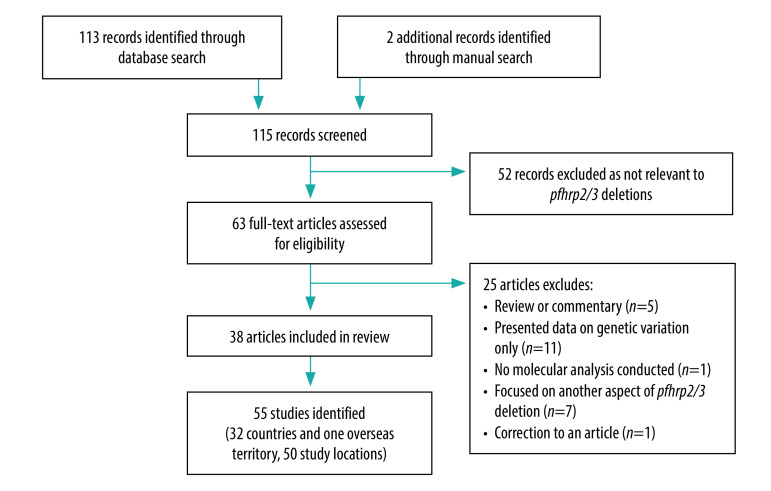
Flowchart of the literature search in the systematic review of *Plasmodium falciparum pfhrp2/3* gene deletions

Note: One article reported samples collected from three countries but did not present the results separately, so it is presented as one study.

After screening 115 articles, we included 38 articles in the review (Fig. 1).^11^^–^^20^^,^^24^^–^^50^ Within the articles we identified 55 distinct studies conducted in 32 countries and one territory in the regions of Africa, Americas, South-East Asia and Eastern Mediterranean (Table 2; available at: http://www.who.int/bulletin/volumes/98/8/20-250621).

**Table 2:** Studies reporting *pfhrp2 and pfhrp3* gene deletions and derived prevalence estimates by region in the systematic review of *Plasmodium falciparum pfhrp2/3* gene deletions

Region and study	Country or territory	Year of data collection	Study sites	Study design	Sample population^a^	Samples tested^b^	Total no. of *P. falciparum* positive patients^c^	No. (%) of samples with gene deletions			Range of *pfhrp2* deletion prevalence across study sites	Quality score	Study bias score
								*Pfhrp2*	*Pfhrp3*	*Pfhrp2 *and* 3*			
**Africa**													
Koita et al., 2012^41^	Mali	1996	Sirakoro, Bancoumana, Doneguebougou, Bamako	Cross-sectional	Mixed	Discordant	480	10^d^ (2)	NA	NA	NA	3	3
Ramutton et al., 2012^35^	Democratic Republic of the Congo	2005–2010	Kinshasa	Health facility (antimalarial drug trial)	Unrepresentative	Subsample	6^e^	0 (0)	0 (0)	0 (0)	NA	5	2
Ramutton et al., 2012^35^	Gambia	2005–2010	Banjul	Health facility (Antimalarial drug trial)	Unrepresentative	Subsample	2^e^	0 (0)	0 (0)	0 (0)	NA	5	2
Ramutton et al., 2012^35^	Kenya	2005–2010	Kilifi	Health facility (antimalarial drug trial)	Unrepresentative	Subsample	12^e^	0 (0)	0 (0)	0 (0)	NA	5	2
Ramutton et al., 2012^35^	Mozambique	2005–2010	Beira	Health facility (antimalarial drug trial)	Unrepresentative	Subsample	19^e^	0 (0)	0 (0)	0 (0)	NA	5	2
Ramutton et al., 2012^35^	Rwanda	2005–2010	Kigali, Nyanza	Health facility (antimalarial drug trial)	Unrepresentative	Subsample	15^e^	0 (0)	0 (0)	0 (0)	NA	5	2
Ramutton et al., 2012^35^	United Republic of Tanzania	2005–2010	Teule, Korogwe	Health facility (antimalarial drug trial)	Unrepresentative	Subsample	18^e^	0 (0)	0 (0)	0 (0)	NA	5	2
Ramutton et al., 2012^35^	Uganda	2005–2010	Mbarare	Health facility (antimalarial drug trial)	Unrepresentative	Subsample	5^e^	0 (0)	0 (0)	0 (0)	NA	5	2
Wurtz et al., 2013^43^	Senegal	2009–2012	Dakar	Health facility (antimalarial drug trial)	Symptomatic	All	125	3 (2)	16 (13)	NA	NA	5	2
Laban et al., 2015^45^	Zambia	2008–2012	Choma	Cross-sectional	Mixed	All	61	NA	NA	12^f^ (20)	NA	4	1
Amoah et al., 2016^16^	Ghana	2015	Abura Dunkwa, Obom	Malaria screening programme	Mixed	All	288	76 (26)	85 (30)	37 (13)	22–40	6	1
Parr et al., 2017^15^	Democratic Republic of the Congo	2013–2014	Kinshasa, Kwango, Kwilu, Mai-Ndombe, Kongo Central, Equateur, Mongala, Nord-Ubangi, Sud-Ubangi, Tshuapa, Kasai, Kasai-Central, Kasai-Oriental, Lomami, Sankuru, Haut-Katanga, Haut-Lomami, Lualaba, Tanganyka, Maniema, Nord-Kivu, Bas-Uele, Haut-Uele, Ituri, Tshopo, Sud-Kivu	Cross-sectional	Mixed	Discordant	2752^g^	149^h^ (5)^i^	NA	5 (< 1)	0–22	5	3
Beshir et al., 2017^9^	Kenya	2014	Mbita	Cross-sectional in schools (mosquito behaviour study)	Asymptomatic	All	131	8 (6)	1 (1)	0 (0)	NA	6	2
Beshir et al., 2017^9^	Kenya	2007–2008	Kilifi	Health facility	Symptomatic	All	49	1 (2)	1 (2)	0 (0)	NA	4	2
Gupta et al., 2017^17^	Mozambique	2010–2016	Manhiça, Magude	Cross-sectional	Mixed	Discordant	1162	1^j^ (< 1)	0 (0)	0 (0)	NA	6	3
Kozycki et al., 2017^14^	Rwanda	2014–2015	Busogo, Kiribizi, Bukara	Health facility	Symptomatic	Discordant	3291	32^k^ (1)	NA	NA	NA	4	4
Menegon et al., 2017^36^	Eritrea	2013–2014	Gash Barka, Debun	Unknown	Unknown	All	144	14 (10)	62 (43)	13 (9)	9–22	4	2
Ranadive et al., 2017^37^	Eswatini	2012–2014	Lubombo	Health facility	Symptomatic	Discordant	162^c^	0^l^ (0)	1 (1)	0 (0)	NA	4	2
Berhane et al., 2018^20^	Eritrea	2016	Northern Red Sea, Anseba, Gash Barka, Debub	Health facility	Symptomatic	All	50	31 (62)	41 (82)	31 (62)	42–81	7	2
Nderu et al., 2018^39^	Kenya	2007–2016	Busia, Mbita, Nyando, Tiwi, Msambweni	Drug efficacy trial	Symptomatic	All	400	0 (0)	0 (0)	0 (0)	NA	5	2
Owusu et al., 2018^38^	Ghana	2015	Greater Accra, Eastern region	Cross-sectional study among patients attending antiretroviral therapy clinics	Unrepresentative	Discordant	62	6^m^(10)	8 (13)	6 (10)	NA	4	3
Willie et al., 2018^40^	Madagascar	2014–2015	Yurimaguas	Health facility	Symptomatic	All	73	0 (0)	NA	NA	NA	3	2
Funwei et al., 2019^42^	Nigeria	2013–2014	Elata Ibadan	Health facility	Symptomatic	Discordant	340	11n (3)	4 (1)	11 (3)	NA	6	4
Kobayashi et al., 2019^46^	Zambia	2009–2011	Choma	Cross-sectional	Mixed	Discordant	45	3^o^ (7)	NA	0 (0)	NA	5	3
Kobayashi et al., 2019^46^	Zambia	2015–2017	Nchelenge	Cross-sectional	Mixed	Discordant	1144	0^p^ (0)	NA	0 (0)	NA	6	3
**Americas**													
Gamboa et al., 2010^11^	Peru	2003–2007	Iquitos area, Loreto, Amazonas, Cajamarca	Health facility	Unknown	All	148	61 (41)	103 (70)	31 (22)	36–100	4	2
Gamboa et al., 2010^11^	Peru	2007	Iquitos	Active case detection survey	Symptomatic	All	9	8 (90)	6 (67)	4 (44)	NA	7	2
Houzé et al., 2011^25^	Brazil	2011	Amazon region	Case study	Symptomatic	Discordant	1	1^q^	1^q^	1^q^	NA	5	4
Maltha et al., 2012^12^	Peru	2010–2011	Iquitos area	Health facility, active case detection	Symptomatic	All	74	19 (26)	34 (44)	19 (26)	NA	6	2
Akinyi et al., 2013^13^	Peru	1998–2001	Loreto, Piura	Unknown	Symptomatic	All	92^c^	19 (21)	NA	NA	0–36	2	2
Akinyi et al., 2013^13^	Peru	2003–2005	Iquitos	Unknown	Symptomatic	All	96^c^	39 (41)	NA	NA	NA	2	2
Trouvay et al., 2013^29^	French Guiana	2009	St Luarent du Maroni, Cayenne, St Georges de l’Oyapack, Saul, Antecume Pata	Unknown	Symptomatic	All	140^c^	0 (0)	4 (3)	0 (0)	NA	2	2
Trouvay et al., 2013^29^	French Guiana	2010–2011	Cayenne Hospital	Health facility survey	Symptomatic	All	81	0 (0)	6 (7)	0 (0)	NA	5	2
Abdallah et al., 2015^32^	Honduras	2008–2009	Puerto Lempira	Health facility (antimalarial drug trial)	Symptomatic	All	68^c^	0 (0)	30 (44)	0 (0)	NA	2	2
Akinyi Okoth et al., 2015^31^	Guyana	2009–2011	Georgetown (Cuyuni-Mazaruni, Potaro-Siparuni)	Health facility survey	Symptomatic	All	97	0 (0)	0 (0)	0 (0)	NA	3	2
Akinyi Okoth et al., 2015^31^	Suriname	2009–2011	Sipaliwini, Brokopondo	Health facility survey, active case detection	Symptomatic	All	78	11 (14)	3 (4)	2 (3)	0–48	3	2
Baldeviano et al., 2015^33^	Peru	2010–2012	Tumbes	Health facility during malaria outbreak	Symptomatic	All	54	54 (100)	NA	NA	NA	2	2
Murillo Solano et al., 2015^26^	Colombia	2008–2009	Cordoba, Narino, Valle del Cauca,	Unknown	Symptomatic	All	75	4 (5)	40 (53)	4 (5)	0–33	6	2
Murillo Solano et al., 2015^26^	Colombia	1999–2007	Amazonas, Guaviare, Meta	Epidemiological studies	Symptomatic	All	25	14 (56)	12 (48)	9 (36)	0–67	4	2
Sáenz et al., 2015^28^	Ecuador	2012–2013	Esmereldas	Malaria outbreak surveillance	Symptomatic	All	32	1 (3)	1 (3)	1 (3)	NA	4	2
Dorado et al., 2016^27^	Colombia	2003–2010	Antiquia, Amazonas, Guaviare, Narino, Choco, Cauca, Valle	Unknown	Symptomatic	All	253	15 (6)	106 (42)	15 (6)	0–54	4	2
Dorado et al., 2016^27^	Colombia	2011–2012	Antiquia, Amazonas, Guaviare, Narino, Choco, Cauca, Valle	Health facility survey	Symptomatic	All	112	0 (0)	51 (42)	0 (0)	NA	6	2
Okoth et al., 2016^34^	Peru	2013	Cusco	Outbreak surveillance	Symptomatic	All	4	4 (100)	4 (100)	4 (100)	NA	4	2
Rachid Viana et al., 2017^24^	Bolivia (Plurinational State of)	2010–2012	Beni department	Health facility survey	Symptomatic	All	25^c^	1 (4)	17 (68)	0 (0)	NA	3	2
Rachid Viana et al., 2017^24^	Brazil	2010–2012	Acre, Para, Rondonia	Health facility survey	Symptomatic	All	198	27 (14)	71 (36)	43 (23)	0–32	4	2
Fontecha et al., 2018^30^	Guatemala	2015	Escuintla	Malaria surveillance survey	Symptomatic	All	21	3 (14)	19 (91)	3 (14)	NA	4	2
Fontecha et al., 2018^30^	Honduras	2011–2017	Gracias a Dios, Colon, Antantida, Cortes, Islas de la Bahia	Health facility survey for drug resistance	Symptomatic	All	52	13 (25)	50 (96)	13 (25)	0–40	4	2
Fontecha et al., 2018^30^	Nicaragua	2015	North Atlantic Autonomous Region	Malaria surveillance survey	Symptomatic	All	55	17 (31)	48 (87)	11 (20)	NA	4	2
**South-East Asia**													
Kumar et al., 2013^49^	India	2010	Chhattisgarh	Unknown	Symptomatic	All	48^c^	2 (4)	2 (4)	2 (4)	NA	6	2
Li et al., 2015^48^	China–Myanmar border, Thailand^r^	2011–2012	China, Myanmar border and Tak province, Thailand	Mass blood survey, unknown	Unknown	All	97	4 (4)	3^s^ (3)	3 (3)	NA	5	2
Bharti et al., 2016^18^	India	2014	Odisha, Chhattisgarh, Jharkhand, Madhya Pradesh, Maharashtra, Rajasthan, Gujarat, Tripura	Health facility	Symptomatic	Discordant	1521	36^t^ (2)	27 (2)	25 (2)	0–25	6	4
Nima et al., 2017^47^	Bangladesh	2013	Sylhet	Case study	Symptomatic	Discordant	1	1^q^	1^q^	1^q^	NA	5	4
Pati et al., 2018^50^	India	2013–2016	Odisha	Cross-sectional	Symptomatic	Discordant	384	38^u^ (10)	24 (6)	17 (4)	8–14	7	4
**Eastern Mediterranean**													
Atroosh et al., 2015^19^	Yemen	2014	Hodeidah, Al-Mahwit	Active case detection	Symptomatic	All	189	9 (5)	NA	NA	NA	4	2
Mussa et al., 2019^44^	Sudan	Unrepresentative	Omdurman	Health facility	Symptomatic	All	26	9 (35)	NA	NA	NA	2	2

DNA: deoxyribonucleic acid; HRP2: histidine-rich protein 2; NA: not applicable; PCR: polymerase chain reaction.

^a^ Symptomatic: only symptomatic people tested; Mixed: mix of symptomatic and asymptomatic people tested; Asymptomatic: only asymptomatic people tested; Unrepresentative: a subset of people not representative of the population were tested; Unknown: not reported.

^b^ All: all samples underwent molecular analysis; Discordant: only discordant samples tested; Subsample: another subset of samples tested.

^c^ As microscopy was not performed, we used the number of *P. falciparum* positive cases by PCR as the denominator.

^d^ Only 22 samples which were rapid diagnostic test-negative and microscopy-positive samples were analysed for *pfhrp2* deletion.

^e^ Samples testing positive by *P. falciparum*-specific lactate dehydrogenase rapid diagnostic test and confirmed by PCR. Only those samples with the lowest level of HRP2 were analysed with molecular methods.

^f^ Only the HRP-leader sequence shared by both *pfhrp2* and *pfhrp3* genes was tested, so we could not break down results by *pfhrp2* or *pfhrp3* genes.

^g^ Microscopy-positive and -negative samples were analysed. PCR-positive samples are included here.

^h^ Only 783 samples which were rapid diagnostic test-negative and PCR-positive were analysed for *pfhrp2* deletion.

^i^ Results differ from those presented in the article as we presented unweighted results.

^j^ Only 69 samples which were rapid diagnostic test-negative and microscopy-positive were analysed for *pfhrp2* deletion.

^k^ Only 138 samples which were rapid diagnostic test-negative and microscopy-positive were analysed for *pfhrp2* deletion.

^l^ Only nine samples which were rapid diagnostic test-negative and quantitative PCR-positive with a parasite counts > 100 per μL were analysed for *pfhrp2* deletion.

^m^ Only eight samples which were rapid diagnostic test-negative and PCR positive were analysed for *pfhrp2* deletion.

^n^ Only 66 samples which were rapid diagnostic test-negative and microscopy- or PCR-positive were analysed for *pfhrp2* deletion.

^o^ Only eight samples which were rapid diagnostic test-negative and quantitative PCR-positive with *pfldh* DNA concentration > 0.0001 ng per µL *P. falciparum* DNA were analysed for *pfhrp2* deletion.

^p^ Only 28 samples which were rapid diagnostic test-negative and microscopy-positive with *pfldh* DNA concentration > 0.0001 ng per µL *P. falciparum* DNA were analysed for *pfhrp2* deletion.

^q^ We did not present prevalence for case studies.

^r^ Article reported collection of samples from three countries but did not present results separately, so the results have been presented here as one study.

^s^ 97 samples were tested for *pfhrp2* deletion, however only the four negative samples were analysed for *pfhrp3* deletion. We present results out of the 97 samples analysed.

^t^ Only 50 samples which were rapid diagnostic test-negative and microscopy-positive were analysed for *pfhrp2* deletion.

^u^ Only 58 samples which were rapid diagnostic test-negative and microscopy-positive were analysed for *pfhrp2* deletion.

Notes: We calculated the prevalence of gene deletions using all *Plasmodium falciparum*-positive samples as the denominators. All studies used microscopy with PCR confirmation, except where indicated. In some cases, only rapid diagnostic test-negative, microscopy-positive samples or rapid diagnostic test-negative, PCR-positive samples were analysed for *pfhrp2* gene deletion, as indicated in footnotes above. We scored study quality from 1 (lowest) to 7 (highest), as described in Box 1, and study bias from 1 (lowest) to 4 (highest).

### Study characteristics

The included studies showed substantial differences in study design, laboratory methods and data reporting. 

#### Sample populations

The number of samples tested for *pfhrp2* ranged from 1 to 783, while the denominator of *P. falciparum*-positive samples ranged from 1 to 3291 (Table 2). Out of the 55 studies, 36 (65%) analysed blood samples only from people with symptoms of malaria, as part of a prospective or retrospective survey including unbiased cohorts. Samples in these studies were collected from suspected malaria patients presenting to health facilities or through active case detection. Eight studies (15%) included samples from asymptomatic and symptomatic people as part of cross-sectional surveys or malaria screening programmes, while eight other studies (15%) used samples from an unrepresentative sample of participants and three studies (5%) did not specify the symptom status of the participants. One study collected samples from patients with severe malaria only, while one study collected equal numbers of samples from human immunodeficiency virus-positive and -negative children.

In 35 studies (64%) all samples underwent *pfhrp2/3* genotyping. Thirteen studies (24%) genotyped discordant samples only. Of these, nine studies analysed only microscopy-positive and HRP2-rapid diagnostic test-negative samples (of which two were case studies including only one sample), while four studies genotyped only samples which were negative by HRP2-rapid diagnostic test and positive by PCR. One article reporting seven studies only genotyped samples showing the lowest HRP2 concentrations by enzyme-linked immunosorbent assay. 

#### Study procedures

Only three studies (5%) fulfilled all seven criteria for quality of procedures (Box 1). While the number of *P. falciparum*-positive samples was based on microscopy- and PCR-positive results in 45 studies (82%), in nine studies (16%) the denominator was based on PCR results alone, and in one study (2%) it was based on *P. falciparum*-specific lactate dehydrogenase-based rapid diagnostic tests and confirmed by PCR. The presence of *P. falciparum* was confirmed most commonly by amplification of the multi-copy *18SrRNA* gene. Thirty-four studies (62%) analysed samples from dried blood spots, 13 (24%) used venous blood, seven (13%) used a combination of both and one study (2%) did not provide information on sample type. Forty-six studies (84%) conducted molecular analysis to determine *pfhrp3* deletion. One of these studies only genotyped *pfhrp3* deletions among samples found to be *pfhrp2-*negative.

Twenty-one studies (38%) did not amplify any other single-copy genes while 13 studies (24%) amplified one other and 21 studies (38%) amplified at least two other single-copy genes. To rule out negative *pfhrp2/3* PCR results being due to parasite density below the limit of detection of the assay, only samples which were positive by other single-copy genes and failed to amplify the *pfhrp2/3* gene were considered to be *pfhrp2/3*-deleted in the 21 studies which conducted this analysis. The most commonly selected genes for confirmation were *P. falciparum* merozoite surface proteins 1 and 2, and glutamate-rich protein. One study confirmed *pfhrp2* deletion by testing for *pfhrp3*. However, while parasite density was measured in 36 studies, only five studies used these results when determining if a sample was *pfhrp2/3*-negative. In three studies only samples above a chosen parasite density or deoxyribonucleic acid (DNA) concentration were tested for *pfhrp2*, while in one study all samples below 5 parasites per µL of blood were discounted and in one study samples were only included in the original study if they were above 2000 parasites per µL of blood. 

Twenty-nine studies (53%) amplified both exons 1 and 2 of the *pfhrp2* gene, while 26 studies (47%) amplified only exon 2 and 28 studies (51%) amplified the flanking genes of *pfhrp2*. The studies that amplified exon 1 were not necessarily those that amplified flanking genes, with 19 studies (35%) amplifying both exon1 and flanking genes.

### Prevalence estimates 

The derived prevalence of *pfhrp2* gene deletions in the 55 studies ranged from 0% to 100% (Table 2). Although we present the overall results by study, 14 studies were conducted over many sites and showed geographically heterogenous results. Further details about the results presented by region are provided in the data repository.^51^

**Fig. 2:**
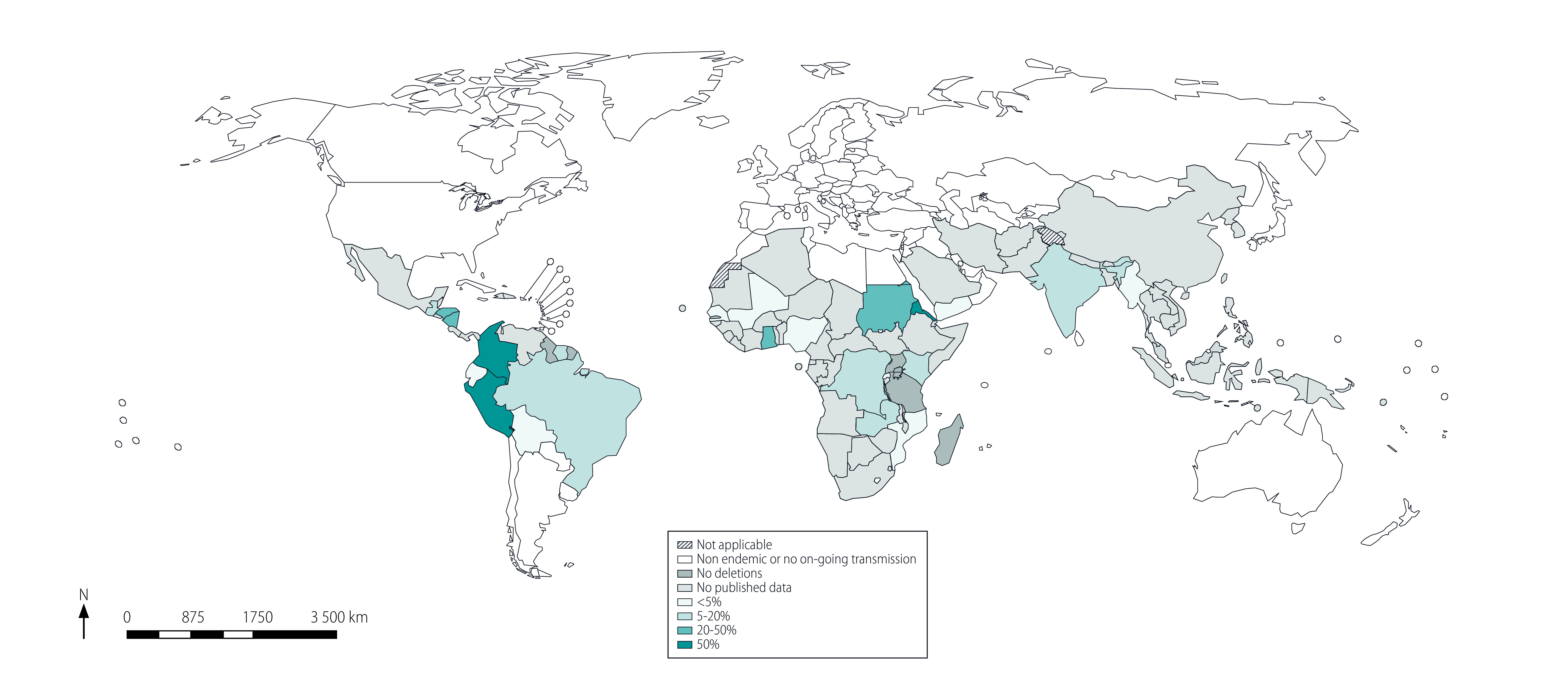
Geographical distribution of highest prevalence estimates for *Plasmodium falciparum pfhrp2* gene deletions by study among patients tested at the country level

Notes: We calculated the prevalence of *pfhrp2* gene deletions using all *Plasmodium falciparum*-positive samples as the denominators by country. We used the highest prevalence estimates for each study.

**Fig. 3:**
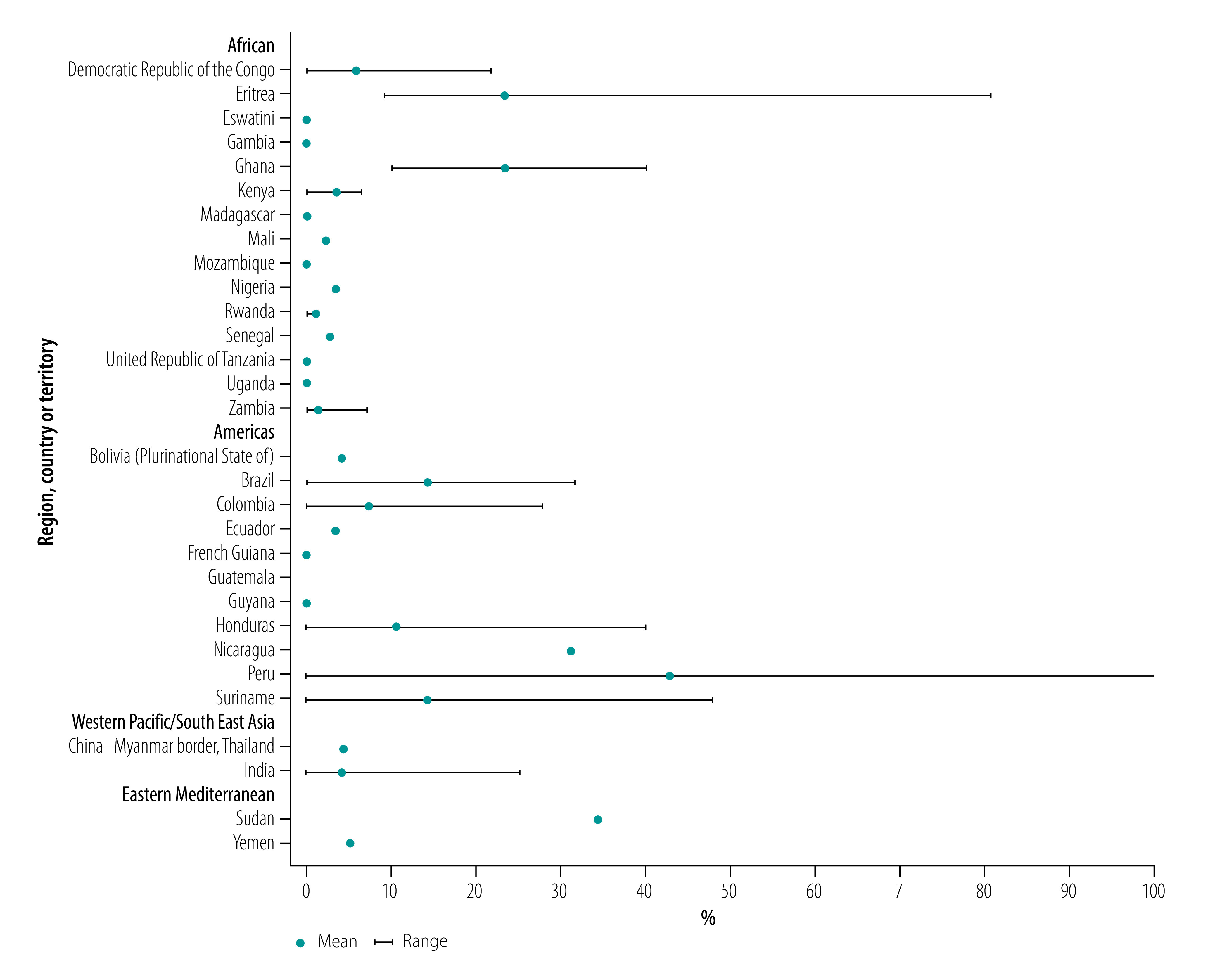
Weighted average prevalence estimates for *Plasmodium falciparum pfhrp2* gene deletions among patients tested by country

Notes: the prevalence of *phfrp2* gene deletions was calculated using the number of *Plasmodium falciparum*-positive samples as the denominator. In countries with multiple studies prevalence estimates were weighted based on the number of patients tested. Countries with only one study have no bar shown. Cases studies and a study from Zambia that tested for *pfhrp2*-leader sequences are not included.

In Fig. 2 we mapped the geographical distribution of the highest derived prevalence estimate of *pfhrp2* gene deletions by study for each country. The highest derived prevalence was above 50% in Colombia, Eritrea and Peru. Fig. 3 plots the weighted average prevalence of *pfhrp2* gene deletions for each country and the range by study sites. The weighted average prevalence ranged from 0% to 43%. Average prevalence above 20% was found in Eritrea, Ghana, Nicaragua, Peru and Sudan.

We plotted the prevalence of *pfhrp2*/3 gene deletions by sample size (available in the data repository).^52^ Five studies had a sample size over 1000, while 36 had sample sizes smaller than 100. All seven studies reporting greater than 50% prevalence of *pfhrp2* deletions had a sample size of fewer than 55. Scatter plots of prevalence against time are available in the data repository.^53^

### Risk of bias

Table 2 shows the bias scores of the prevalence estimates from the true prevalence of *pfhrp2/3* gene deletions in the population. Six studies (11%) had a bias score of four while two (4%) had a bias score of one. 

## Discussion

We found that mutant parasites have been reported from all major malaria-endemic areas, in asymptomatic and symptomatic *P. falciparum*-positive patients. However, our results also confirm that the full extent of the threat has not yet been characterized. The limited number of well conducted prevalence surveys in malaria-endemic countries indicate geographical variability in the prevalence of mutations in the *pfhrp2* and *pfhrp3* genes and do not completely illuminate the factors driving these differences.

The study has limitations. Although we included only published articles, we were aware of other abstracts and doctoral theses for which relevant data on methods were not available. For manuscripts included in the review, we contacted authors to obtain information not included in the manuscripts; this was not always possible, however, and we therefore occasionally made assumptions about the methods. Survey design and sample populations varied greatly across the included studies. Most studies were not purposely designed for investigating the prevalence of gene deletions and relied on convenience sampling or on secondary analyses of existing specimens. These shortcomings limit our ability to draw conclusions that can inform the use of rapid diagnostic testing, but rather provides guidance for future surveys.

Reconciling the different populations and sample sizes across studies is challenging. First, studies of asymptomatic and symptomatic patients require different interpretations and are difficult to integrate. Samples from asymptomatic patients may have lower parasite densities, resulting in less DNA target for amplification and potentially greater risk of falsely reporting *pfhrp2* deletions. This risk is especially high when the investigation does not include amplification of other single-copy genes or does not quantify parasite DNA. Furthermore, little is known about the effect of *pfhrp2/3* gene deletions on the virulence of malaria infection. If *pfhrp2/3* deletions are associated with less virulent infections, there could be a difference in prevalence between symptomatic and asymptomatic infections. We found numerous studies with low sample sizes which may not be representative of the true prevalence of deletions in a population or country.

Second, different selection criteria for *pfhrp2/3* genotyping (all malaria suspects or only those with discordant HRP2-based rapid diagnostic test and microscopy and/or PCR results) result in the use of different numerators and denominators for prevalence estimation across studies. Analysis limited to deletions found among discordant samples leads to a higher prevalence of gene deletions being reported. To improve consistency in calculating the prevalence of *pfhrp2-*negative mutants, we used the total number of samples with confirmed *pfhrp2* gene deletions by species-specific PCR as the numerator and the total cohort number of *P. falciparum*-positive samples by microscopy and/or PCR as the denominator. The WHO-recommended approach of testing only a subset of high-risk (discordant) samples is a more economical way of monitoring the prevalence of gene deletion and targets clinically significant deletions that cause negative test results. WHO recommends using non-exclusive HRP2-based rapid diagnostic tests when the prevalence of *pfhrp2/3* gene deletions causing false-negative test results is greater than 5%.^22^ Most studies included in this review did not allow us to determine if the WHO threshold had been exceeded. It is well acknowledged that the WHO approach may underestimate the prevalence of *pfhrp2* deletions in the parasite population. Samples that are *pfhrp2-*negative and *pfhrp3*-positive are not likely to be flagged as high risk or discordant due to the cross-reactivity between HRP2 and HRP3 proteins on many rapid diagnostic test brands. However, this concern does not pose an immediate threat to patients.^54^ Ideally, all *P. falciparum*-positive samples should be used as the denominator, determined either by microscopy or a good quality rapid diagnostic test for detecting *P. falciparum*-specific lactate dehydrogenase.

The study bias scores show the potential bias of the prevalence estimates from the true prevalence of *pfhrp2/3* gene deletions in the population, but not necessarily the bias of deletions causing false-negative results (which is more important for determining the effect on malaria case management). Ultimately, high-throughput screening options could become more readily available and more commonly used. If so, the true prevalence of *pfhrp2* gene deletions could be determined by molecular testing of all people with suspected malaria regardless of rapid diagnostic test or microscopy results, and those samples confirmed to have *pfhrp2* deletions used as the numerator.

Third, recent modelling suggests that the likelihood of finding *pfhrp2/3* deletions can vary during the malaria transmission season due to changes in the transmission intensity and multiplicity of infection, whereby a person can be infected with multiple *P. falciparum* strains.^55^ Co-infection with *pfhrp2/3*-negative- and wild-type parasites can prevent detection of gene deletions using current laboratory techniques, leading to an underestimation of the prevalence of *pfhrp2/3*-negative mutants. Time of year and relation to the transmission season is rarely described in published reports. A publicly available database using prediction models could be useful to help determine the optimal time in the transmission season to conduct a gene deletion survey.^56^

Due to the small number of studies, differing populations and often small samples sizes of the reviewed studies, it is difficult to draw robust conclusions on the prevalence of *pfhrp2/3* gene deletions in specific areas or to perform meta-analysis from these data. The implementation of more large-scale, robust surveys would enable a better understanding of if, and at what rate, these mutations are increasing in a given area, and would allow for meta-analysis.

Identifying the prevalence of *pfhrp2/3* deletion mutations requires synthesis of several lines of evidence and study procedures that include proper performance of rapid diagnostic tests and careful genotyping methods. While most studies in this review followed some components of published criteria to classify a sample as *pfhrp2*-deleted,^21^ only 3 (5%) of the studies followed the seven recommended criteria proposed in this review. One specific challenge for molecular analyses of *pfhrp2/3* is using the absence of amplified products as the indicator of interest, rather than the presence of amplified products. Rigorous methods and appropriate controls must be used to ensure the presence of non-degraded, amplifiable parasite DNA and the lack of amplicon contamination. Improving the accuracy of survey outcomes requires novel molecular-based technology and methods that could detect *pfhrp2/3* gene deletions more reliably and efficiently and detect *pfhrp2/3* deletions in samples with mixed infections (such as quantitative-PCR and whole genome sequencing). Not all malaria-endemic countries have the capacity to conduct molecular analysis to a high standard, and establishing such capacity is challenging and costly. In addition, the sensitivity and specificity of PCR assays can be affected by the protocol used, potentially resulting in variations in the results across laboratories following different procedures. For example, lowering the elongation temperature on *pfhrp2* assays improved the limit of detection of many previously published assays.^57^ WHO has established a network of laboratories capable of conducting this analysis to ensure that samples from prevalence surveys can be performed quickly and procedures harmonized across laboratories.^22^

Just over half of the studies amplified both exon 1 and 2 of the *pfhrp2* gene, while the rest amplified only exon 2. While the chromosomal break points could theoretically be anywhere within the *pfhrp2* gene, it appeared that most samples from Eritrea and Peru have observed deletions in both exon 1 and 2 (Qin Cheng, Australian Defence Force Malaria and Infectious Disease Institute, personal communication, 2019). Therefore, whether analysis of exon 2 alone is sufficient to identify most parasites with *pfhrp2* gene deletions requires further analysis of gene deletions from other parasite populations. While not included in the recommendations for *pfhrp2/3* molecular analysis,^21^ analysis of flanking genes can provide additional information on genetic mutations.

Despite the diversity of study approaches, there appear to be areas of high prevalence of *pfhrp2/3* mutant parasites where diagnostic testing based on HRP2 alone would be inadequate. Thus, the need for alternative rapid diagnostic tests is of urgent concern in the Amazon basin and Eritrea, where the prevalence of tests producing false-negative results among symptomatic patients has forced changes in the diagnostic strategy.^58^ Malaria control programmes should remain vigilant for evidence suggesting the presence of *pfhrp2/3* gene deletions. Evidence of false-negative rapid diagnostic tests or confirmed *pfhrp2/3-*negative mutants in neighbouring countries should trigger careful investigation and surveillance. To improve the quality and relevance of surveys for clinical case management, WHO now provides general guidance on when to prioritize surveys for *pfhrp2/3* deletions.^22^ WHO has also developed protocols for guiding survey design, data collection and laboratory methods to determine the prevalence of clinically-relevant *pfhrp2/3* deletions causing false-negative rapid diagnostic tests.^59^ The guidelines aim to ensure that future investigations are implemented to high and comparable standards. Additionally, an up-to-date repository of *pfhrp2/3* deletion studies is maintained on the WHO malaria threat map.^60^

The specific factors that drive the evolution and spread of *pfhrp2/3* mutations are not clear, although mathematical models suggest that selective pressure by HRP2-detecting rapid diagnostic tests over the past decade is likely to have played an important role.^5^ Low malaria transmission and high frequency of people correctly treated on the basis of diagnosis with HRP2-detecting tests have also been identified as key drivers of the selection of *pfhrp2/3*-negative mutants.^61^ Nevertheless, the existence and rising prevalence of *pfhrp2* deletions in Peru,^11^^,^^12^^,^^33^ where HRP2-only methods have never been widely used, along with the local prevalence of *pfhrp3* mutations, confirms that selective treatment based on test results is not the only factor driving the evolution of these parasites.

Due to the global reliance on rapid diagnostic tests for malaria diagnosis, a coordinated, multifaceted response to P. falciparum with *pfhrp2/3* gene deletions is required. This response should include representative studies of the prevalence and distribution of *pfhrp2/3* deletions, more efficient and affordable methods for screening and confirming these deletions, and efforts to standardize and ensure high-quality reporting. Follow-up surveys in areas with documented *pfhrp2/3* deletions will provide insight into the speed at which the mutant parasites are evolving in response to diagnostic pressure and other drivers. Research for the development and commercialization of rapid diagnostic tests based on new or improved non-HRP2 targets is an essential parallel area of work.
